# Hydrolases of the ILR1-like family of Arabidopsis thaliana modulate auxin response by regulating auxin homeostasis in the endoplasmic reticulum

**DOI:** 10.1038/srep24212

**Published:** 2016-04-11

**Authors:** Ana Paula Sanchez Carranza, Aparajita Singh, Karoline Steinberger, Kishore Panigrahi, Klaus Palme, Alexander Dovzhenko, Cristina Dal Bosco

**Affiliations:** 1 Institute of Biology II/Molecular Plant Physiology, Faculty of Biology, Albert-Ludwigs-University of Freiburg, Schänzlestrasse 1, D-79104 Freiburg, Germany; 2 National Institute of Science Education and Research, Institute of Physics Campus, Bhubaneswar, Odisha 751005, India; 3 BIOSS Centre for Biological Signalling Studies, University of Freiburg, 79104 Freiburg, Germany; 4 Freiburg Institute for Advanced Sciences (FRIAS), University of Freiburg, 79104 Freiburg, Germany; 5 Centre for Biological Systems Analysis (ZBSA), University of Freiburg, 79104 Freiburg, Germany

## Abstract

Amide-linked conjugates of indole-3-acetic acid (IAA) have been identified in most plant species. They function in storage, inactivation or inhibition of the growth regulator auxin. We investigated how the major known endogenous amide-linked IAA conjugates with auxin-like activity act in auxin signaling and what role ILR1-like proteins play in this process in Arabidopsis. We used a genetically encoded auxin sensor to show that IAA-Leu, IAA-Ala and IAA-Phe act through the TIR1-dependent signaling pathway. Furthermore, by using the sensor as a free IAA reporter, we followed conjugate hydrolysis mediated by ILR1, ILL2 and IAR3 in plant cells and correlated the activity of the hydrolases with a modulation of auxin response. The conjugate preferences that we observed are in agreement with available *in vitro* data for ILR1. Moreover, we identified IAA-Leu as an additional substrate for IAR3 and showed that ILL2 has a more moderate kinetic performance than observed *in vitro*. Finally, we proved that IAR3, ILL2 and ILR1 reside in the endoplasmic reticulum, indicating that in this compartment the hydrolases regulate the rates of amido-IAA hydrolysis which results in activation of auxin signaling.

The cellular pool of the plant hormone auxin is represented by free indole-3-acetic acid (IAA), IAA precursors and IAA conjugated forms. Conjugated forms of IAA account for a significant amount of total IAA within most plant tissues and they include amide-linked conjugates such as amino acids and peptides and ester-linked conjugates such as *myo-*inositol and *myo*-inositol-sugars^1^,^2^. Ester-linked conjugates are found in endosperm tissues of monocots and dicots, whereas amide-linked IAA-l-amino acids conjugates (IAA-aa) predominate in mature dicot seeds and light grown vegetative tissues of most plants, both monocots and dicots^3^. Various IAA-aa conjugates have been identified in different plant species and include IAA-leucine (IAA-Leu), IAA-alanine (IAA-Ala), IAA-aspartate (IAA-Asp), IAA-glutamate (IAA-Glu), and IAA-tryptophan (IAA-Trp)^4^,^5^. The occurrence of other conjugates such as IAA-valine (IAA-Val) and IAA-phenylalanine (IAA-Phe) has been postulated based on the identification of oxidative metabolites in Arabidopsis^6^. The different IAA-aa conjugates have been implicated in a variety of biological processes. For example, IAA-Glu and IAA-Asp, which are readily synthesized upon incubation of plants with high dosage of IAA^4^, are considered precursors for auxin degradation. IAA-Trp functions as an endogenous inhibitor which interferes with several physiological responses to auxin^7^. IAA-Trp requires the auxin receptor TIR1 for full activity though reaction pathways are not yet elucidated^7^. Other conjugates such as IAA-Ala, -Leu, -Phe, -Val are biologically active and mimic the effect of free IAA in inducing plant developmental responses like root and hypocotyl elongation inhibition^8^.

Early studies showed that the biological activity of IAA-aa conjugates was related to the amount of free IAA detected in the stem tissue resulting from their hydrolysis^9^. This demonstrated, for the first time, a direct link between the activity of the conjugates and their hydrolysis.

Mutant screens based on reduced sensitivity to biologically active IAA-aa in root growth inhibition assays led to the identification of a specific group of amidohydrolases^8^,^10^. IAA-Leu-resistant1 (*ilr1*) was the first mutant to be identified; the gene encodes an amidohydrolase with high affinity for IAA-Leu and IAA-Phe^8^. In Arabidopsis, the ILR1-like (ILL) family consists of 7 members: ILR1, ILL1, ILL2, ILL3, IAR3 (ILL4), ILL5 and ILL6. The best characterized are ILR1, ILL1, ILL2, and IAR3 for which IAA-amino acids cleavage activity and substrate specificity have been shown by *in vitro* enzymatic assays^10^,^11^. IAR3 and ILL2 show highest catalytic activity with IAA-Ala as a substrate, while ILR1 is most efficient in hydrolyzing IAA-Leu and IAA-Phe. ILL3 and ILL6 show no activity on IAA-aa *in vitro*^10^ or very little activity^11^. *ILL5* is an apparent pseudogene^10^,^11^. IAA-Asp and IAA-Glu are not efficiently hydrolyzed by any of the Arabidopsis amidohydrolases^10^. Single mutants of the ILR-1 like family such as *ilr1, ill2* and *iar3*, display a reduced sensitivity to biologically active IAA-aa conjugates, while plants overexpressing the hydrolases show higher sensitivity to certain IAA-aa conjugates^12^. The triple mutant *ilr1iar3ill2* is less sensitive to IAA-Phe and IAA-Ala and essentially insensitive to IAA-Leu^13^. These genetic data correlate with hydrolysis rates for each IAA-aa conjugate reported from *in vitro* enzymatic assays^10^, suggesting that the hydrolase activity of ILR1-like proteins is directly linked to the biological activity of the IAA-aa conjugates.

This study evaluates how the major endogenous IAA-aa conjugates with auxin-like activity act in auxin signaling and how this activity is modulated by the amidohydrolases. We applied a genetically encoded auxin sensor which is based on the TIR1-mediated auxin-dependent degradation of Aux/IAAs^14^ and showed that the biological activity of IAA-Ala, IAA-Leu and IAA-Phe is, at least in part, mediated by the TIR1-dependent auxin signaling pathway. Additionally, by using the sensor as a reporter of free IAA, we investigated the hydrolysis activity of members of ILR1-like family in a single plant cell system. Thereby we positively correlated conjugate preference for ILR1 and IAR3 with *in vitro* data^10^. Furthermore, we identified IAA-Leu as an additional substrate for IAR3 and found a different conjugate preference and a more moderate kinetic performance for ILL2. Finally, we experimentally showed that IAR3, ILL2 and ILR1 are localized in the endoplasmic reticulum (ER). This defines the ER as the compartment where the amidohydrolases modulate the rate of IAA-aa hydrolysis which results in activation of auxin signaling.

## Results and Discussion

### Correlating amide-conjugates IAA-Ala, IAA-Leu and IAA-Phe with auxin signaling

IAA-aa conjugates IAA-Ala, IAA-Leu and IAA-Phe mimic the effect of free IAA in inducing plant developmental responses in Arabidopsis^13^. Genetic and biochemical studies indicate a direct relationship between the biological activity of these conjugates and their hydrolysis rates mediated by amidohydrolases from the ILR1-like family. The underlying molecular basis of IAA-like activity however has not yet been elucidated. To address this question, we investigated how IAA-aa and their hydrolysis act in term of auxin perception and activation of auxin-signaling pathway.

Auxin signaling is initiated through binding of IAA to the TRANSPORT INHIBITOR RESPONSE1/AUXIN SIGNALING F-BOX PROTEIN (TIR1/AFB) and AUXIN/INDOLE ACETIC ACID (Aux/IAA) protein co-receptors and the consequent targeting of the Aux/IAA proteins for degradation^15^,^16^,^17^. Upon Aux/IAA degradation, repression of AUXIN RESPONSIVE FACTORS (ARFs) transcription factors is released and transcription of auxin-regulated genes takes place, thereby initiating auxin signaling. In light of this, we investigated how biologically active IAA-aa conjugates are involved in the TIR1/AFBs-Aux/IAAs-ARFs pathway by employing a genetically encoded ratiometric auxin sensor^14^. The functional principle of this sensor is based upon the auxin-dependent formation of a TIR1/AFB-Aux/IAA-reporter like complex. The sensor comprises two modules: an auxin responsive module and an auxin-insensitive module. The auxin responsive module consists of firefly luciferase translationally fused with the conserved degron motive of Aux/IAAs. The auxin-insensitive module consists of renilla luciferase and serves as normalization of response. Thus, upon expression of the sensor in plant cells, auxin-dependent degradation of the sensor is monitored as a decrease in firefly (responsive module) relative to renilla (normalization module) luminescence. Notably, this degradation-based sensor is different from widely used *DR5*- based auxin inducible reporters whose functional principle relies on ARF’s binding sites and activation of auxin signalling^18^,^19^.

**Figure 1:**
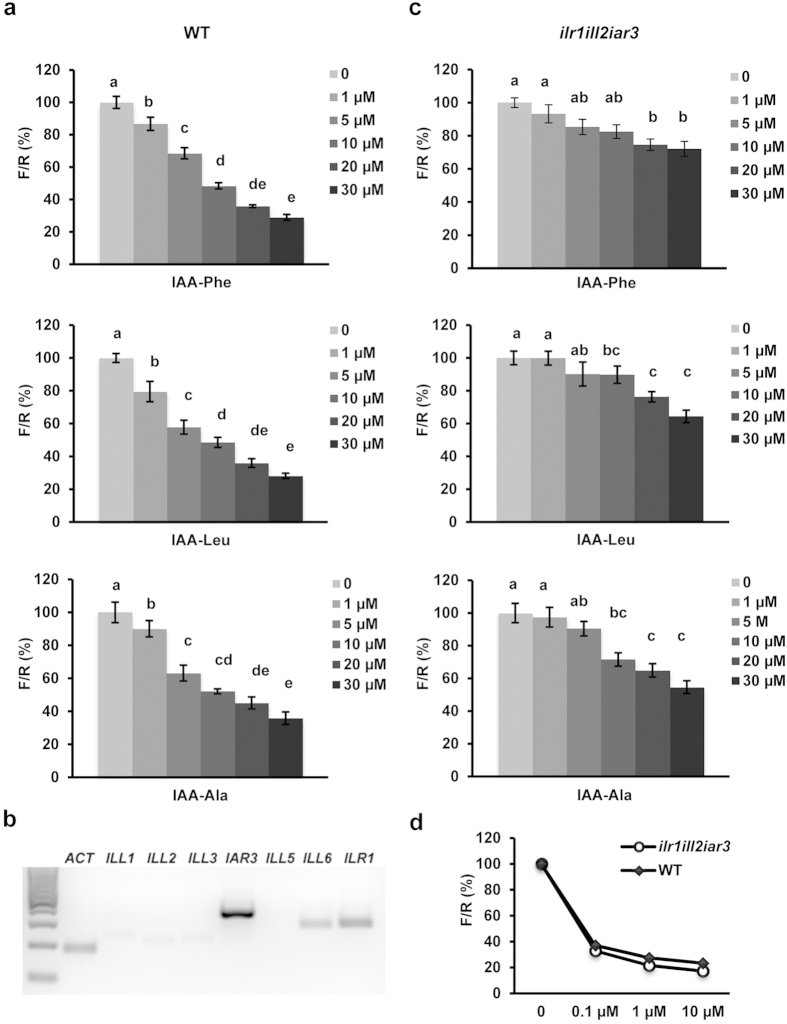
Correlation of amide-conjugates IAA-Ala, IAA-Leu and IAA-Phe with TIR1/AFBs signaling pathway using ratiometric auxin sensor in plant cells. (**a,c,d**) Sensitivity of the auxin sensor towards IAA-Ala, IAA-Leu, IAA-Phe and IAA, in plant cells. Protoplasts from Col-0 wild type (**a**) *ilr1ill2iar3* triple mutant (**c**) or both (**d**) were transformed with the sensor construct. After 20 h incubation in hormone-free medium, 0, 1, 5, 10, 20, 30 μM each of IAA-Ala, IAA-Leu and IAA-Phe (**a,c**) or 0, 0.1, 1, 10 μM IAA (**d**) were added to the samples. After 1 h treatment, the ratiometric determinations of firefly/renilla (F/R) were performed. Results are represented as percentile of the control samples (no auxin treatment). Results are means ± s.e.m. (*n* = 16). Statistical significances for each conjugates are indicated with lower case letters (the same letters indicate no statistical significant difference between the samples, one-way ANOVA, *P* < 0.001). (**b**) Expression analysis of *ILL1, ILL2, ILL3, IAR3, ILL5,* ILL6 and *ILR1* in Arabidopsis Col-0 protoplast-derived cells. *ACTIN* (*ACT*) was included as a positive control.

Previously, it was shown that the sensor enables quantitative monitoring of changes in cellular levels of IAA as well as of synthetic compounds with auxin-like activity^14^. We first explored whether the biologically active IAA conjugates led to TIR1-dependent sensor degradation. Arabidopsis leaf protoplast cells expressing the sensor were incubated with each conjugate at various concentrations for 1 hour and then the ratiometric determinations of firefly and renilla luminescence (F/R) were performed (Fig. 1a). All conjugates induced sensor degradation starting as low as 1 μM and resulted in ~50% of sensor degradation at 10 μM. An even stronger effect was observed at higher concentrations (Fig. 1a). These results indicate that biologically active conjugates may trigger the sensor degradation either by direct binding to TIR1/AFBs and subsequent ubiquitination and degradation of the sensor or it is free IAA, released upon hydrolysis, acting as the effector molecule.

Therefore, to understand whether the sensor degradation observed upon incubation with the IAA conjugates (Fig. 1a) requires the activity of the hydrolases, we analyzed the expression levels of members of the ILR1-like genes in Arabidopsis leaf protoplasts. RT-PCR analysis showed that *ILR1, ILL6* and *IAR3* were expressed in Arabidopsis cells (Fig. 1b). Expression level of *ILL1*, *ILL2* and *ILL3* was at the limit of detection, indicating low abundance of these gene transcripts. *ILL5* could not be amplified. The presence of *ILR1, ILL6* and *IAR3* in protoplasts indicates that the sensor degradation might indeed reflect the accumulation of free IAA in the cells upon conjugate hydrolysis. To validate this suggestion, we investigated mutant plants defective of ILR1, IAR3 and ILL2, the hydrolases which showed the highest *in vitro* hydrolysis performance towards IAA-amido conjugates^13^.

The degradation kinetic analysis of the auxin sensor was compared in WT and *ilr1ill2iar3* triple mutant cells^20^. Impaired hydrolysis resulted in a significant reduction of sensitivity towards the three conjugates (Fig. 1c). Treatment with up to 5 μM of either conjugate resulted in statistically insignificant sensor degradation in *ilr1iar3ill2* cells. At the highest concentration (30 μM), sensor degradation was strongly decreased: 30% vs. 71% (IAA-Phe), 40% vs. 72% (IAA-Leu), and 50% vs. 65% (IAA-Ala) in triple mutant and WT cells, respectively. To test whether auxin response was in general affected in the triple mutant, treatment with free IAA in WT and *ilr1iar3ill2* cells was performed (Fig. 1d). The sensor was degraded in the triple mutant to the same extent as in WT indicating that the TIR1/AFBs pathway is not affected in the triple mutant.

**Figure 2:**
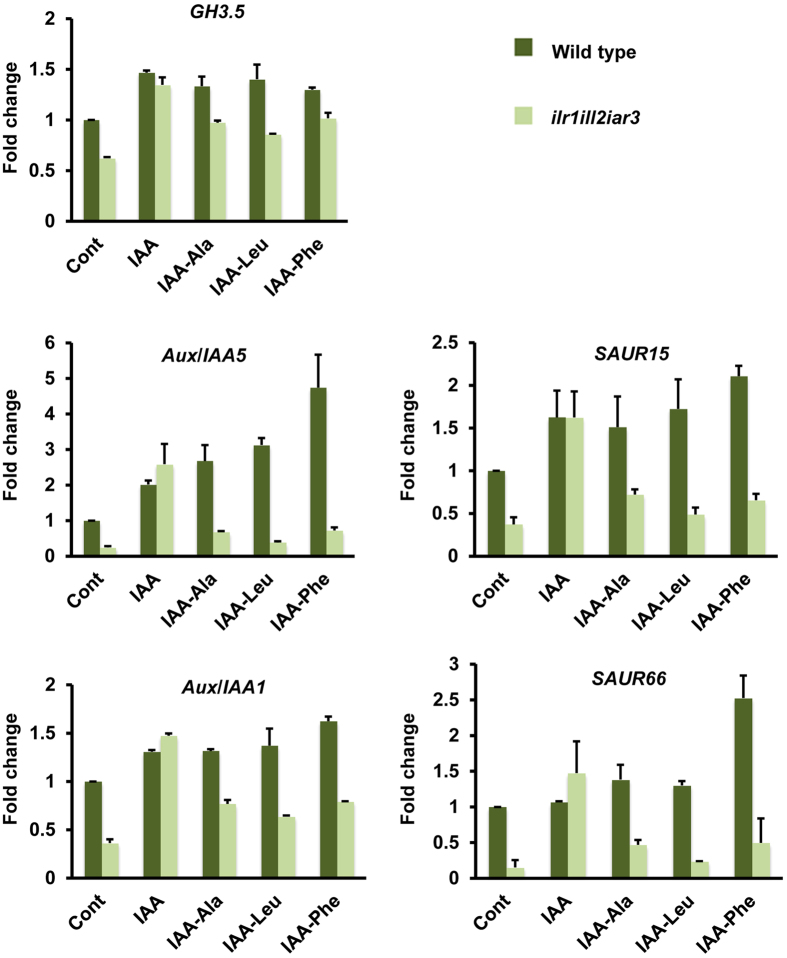
Expression analysis of early auxin induced genes in Arabidopsis Col-0 wild type and *ilr1ill2air3* triple mutant seedlings. The seedlings were incubated for 1 hour in liquid culture medium (CONT), or supplied with 10 μM each of IAA-Ala, IAA-Leu, IAA-Phe or 100 nM IAA. Expression analysis of *Aux*/*IAA1*, *Aux*/*IAA5*, *GH3.5*, *SAUR15*, *SAUR 66* (auxin regulated genes) was performed using quantitative real time PCR. Transcript levels of target genes were normalized to expression of *ACTIN* (housekeeping gene). Expression is shown relative to wild type (CONT). Results are means ± s.e.m. (*n* = 2).

We further analyzed the effect of IAA-aa conjugates and their hydrolysis on transcriptional activation of auxin responsive genes. The best characterized early auxin response genes are represented by three gene families^21^: *Aux/IAAs*, *SMALL AUXIN UP RNAs* (*SAURs*), and *GRETCHEN HAGEN3s* (*GH3s*). We therefore tested the expression of representative members of these gene families, i.e. *Aux*/*IAA1*, *Aux*/*IAA5*, *SAUR15, SAUR66* and *GH3.5*, upon treatment with IAA-aa conjugates in Arabidopsis WT and *ilr1ill2iar3* seedlings. Free IAA was used as a positive control. Quantitative real time PCR (qPCR) analysis (Fig. 2) revealed a lower basal expression level of auxin responsive genes in the triple mutant. This result is in accordance with the developmental phenotypes and a reduced free IAA level measured in *ilr1ill2ir3* triple mutant^13^. Analysis of gene expression upon treatment with free IAA showed similar expression levels of *Aux*/*IAA1*, *Aux*/*IAA5*, *SAUR15, SAUR66* and *GH3.5* in both, triple mutant and WT seedlings. On the other hand, analysis of gene expression upon treatment with IAA-Ala, IAA-Leu and IAA-Phe showed that induction of auxin regulated genes was affected in the mutant (Fig. 2).

Collectively these results indicate that IAA-mediated auxin signaling machinery *per se* is not impaired in the triple mutant and that activation of auxin signaling cascade by IAA-aa conjugates requires the activity of ILR1-like hydrolases.

### Application of the auxin sensor to monitor IAA amide conjugate hydrolysis in plant cells

**Figure 3:**
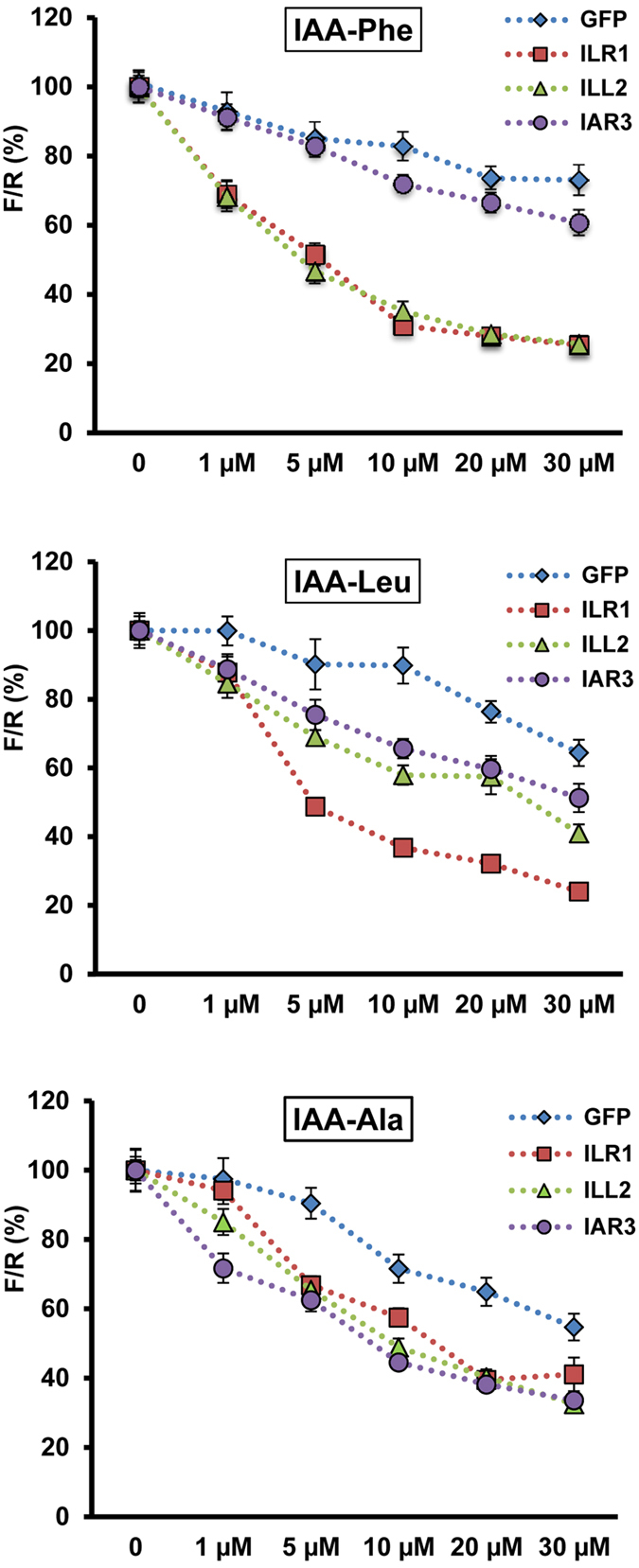
Application of the auxin sensor to monitor the modulation of intracellular auxin pool mediated by ILR1, ILL2 and IAR3, in plant cells. The sensor was co-expressed with either ILR1, or ILL2 or IAR3 in triple mutant *ilr1ill2iar3* protoplasts. GFP was also co-expressed as a control. After 20 h incubation in hormone-free medium, 0, 1, 5, 10, 20, 30 μM each of IAA-Ala, IAA-Leu and IAA-Phe were supplemented to the culture medium. After 1 h treatment, the ratiometric determinations (F/R) were performed. Results are represented as percentile of the control samples (no auxin treatment). Results are means ± s.e.m. (*n* = 16).

The ratiometric auxin sensor assays showed the relevance of the hydrolases to modulate intracellular free IAA levels. Furthermore, these assays provide an *ex vivo* experimental system to study the hydrolase activity, which so far has only been characterized *in vitro*^10^,^11^. Therefore we applied the auxin sensor to study conjugate hydrolysis mediated by ILR1, ILL2 and IAR3 in plant cells. The sensor was co-expressed with ILR1, IAR3, ILL2 or GFP (a protein not involved in auxin metabolism) as a negative control. Comparable expression of the hydrolases was confirmed at transcript and protein levels (Supplementary Fig. S1). To highlight the specific activity of each hydrolase and to minimize the effect of endogenous enzymes, the assays were performed in *ilr1ill2iar3* cells. 20 hours after transformation, the cells were incubated with either IAA-Leu, IAA-Phe, or IAA-Ala, for 1 hour. For all tested conjugates, increased sensor degradation was observed upon over expression of the hydrolases (Fig. 3). However, the conjugate preference and the relative activity differed among the three enzymes. IAA-Phe was hydrolyzed by ILR1 and ILL2. IAA-Leu was preferentially hydrolyzed by ILR1 and to lesser extent by ILL2 and IAR3. IAA-Ala, which retained most activity in the triple mutant cells, was hydrolyzed by all three hydrolases but to lesser extent than IAA-Leu and IAA-Phe. These results show that the three hydrolases are involved in activation of auxin signaling by hydrolysis of IAA-Ala, -Leu and -Phe into biologically active IAA. Particularly, ILR1 is primarily involved in mediating plant responses to IAA-Leu and IAA-Phe, IAR3 to IAA-Ala and IAA-Leu and ILL2 to IAA-Phe and IAA-Ala.

These findings are in positive correlation with physiological assays performed with the hydrolase mutants and partly in agreement with the reported hydrolase activity and substrate specificities observed *in vitro*. For example, ILR1-mediated *in vitro* hydrolysis activity was reported for IAA-Phe, IAA-Leu and IAA-Ala^10^. Similarly, in root elongation inhibition assays on single, double and triple mutants, *ilr1* contributed to resistance to IAA-Leu, IAA-Phe and to a minor extent to IAA-Ala^13^. Our data on ILR1 are in agreement with these results. On the other hand, IAR3 showed *in vitro* hydrolysis activity with IAA-Ala, but no activity with IAA-Leu^10^. Our assays confirmed IAA-Ala as a substrate of IAR3, but also identified IAA-Leu as an additional substrate. This finding can now explain the contribution of *iar3* to IAA-Leu resistance observed in physiological assays when combined with *ilr1ill2* mutant^13^. The least agreement between our and *in vitro* data was found for ILL2. ILL2 showed highest amidohydrolase activity *in vitro,* with the broadest range of substrate specificity and highest hydrolysis activity with IAA-Ala^10^. In our assays, ILL2 showed highest hydrolysis activity with IAA-Phe, with comparable sensor degradation kinetics as ILR1, while IAR3 showed higher hydrolysis activity with IAA-Ala than ILL2. The reasons for the discrepancy between our data and *in vitro* characterization could be diverse. Multiple factors which are absent in standardized *in vitro* conditions might influence the activity of an enzyme *in vivo*. For example, while determining optimal conditions for hydrolysis activity it was shown that different pH and metal ion cofactors influenced the hydrolysis rates^10^. Therefore metal ion homeostasis in the cell may modulate the activity of the hydrolases differently. Accordingly, the *ilr2* mutant, which is resistant to IAA-Phe and IAA Leu, exhibited altered metal transport^22^.

Interestingly, the conjugate preference and a more moderate hydrolysis activity of ILL2 observed in the plant cell assays are in agreement with genetic data. Albeit ILL2 was the most active hydrolases *in vitro*^10^, no *ill2* alleles were isolated in genetic screens for conjugate resistant seedlings (in contrast to *ilr1* and *iar3* alleles)^23^. Additionally, when *ill2* allele was combined in double and triple mutants with *ilr1* and *iar3*, it mainly contributed to resistance to IAA-Phe and to lesser extent to IAA-Ala^13^. Our sensor-based assays also showed that IAA-Ala was the least hydrolyzed by ILR1, IAR3 or ILL2. This suggests that other proteins might be involved in hydrolysis of IAA-Ala. In protoplasts at least one more member of ILR1-like family is expressed: *ILL6* (Fig. 1b). However, ILL6 showed no^10^ or little *in vitro* hydrolysis activity, around 10 fold less than IAR3 with IAA-Ala as a substrate^11^. Higher order hydrolase mutants will help in future to discriminate the contribution of other hydrolases for IAA-Ala sensitivity.

Our experimental approach proved efficient to study the auxin conjugate hydrolase activity in Arabidopsis single cells, thus representing a valuable tool to complement *in vitro* studies. Moreover, this system could be adapted to the analysis of other genes and physiologically active compounds involved in auxin homeostasis and signaling networks in Arabidopsis and other plant species.

### Subcellular localization of ILL2, ILR1 and IAR3

**Figure 4:**
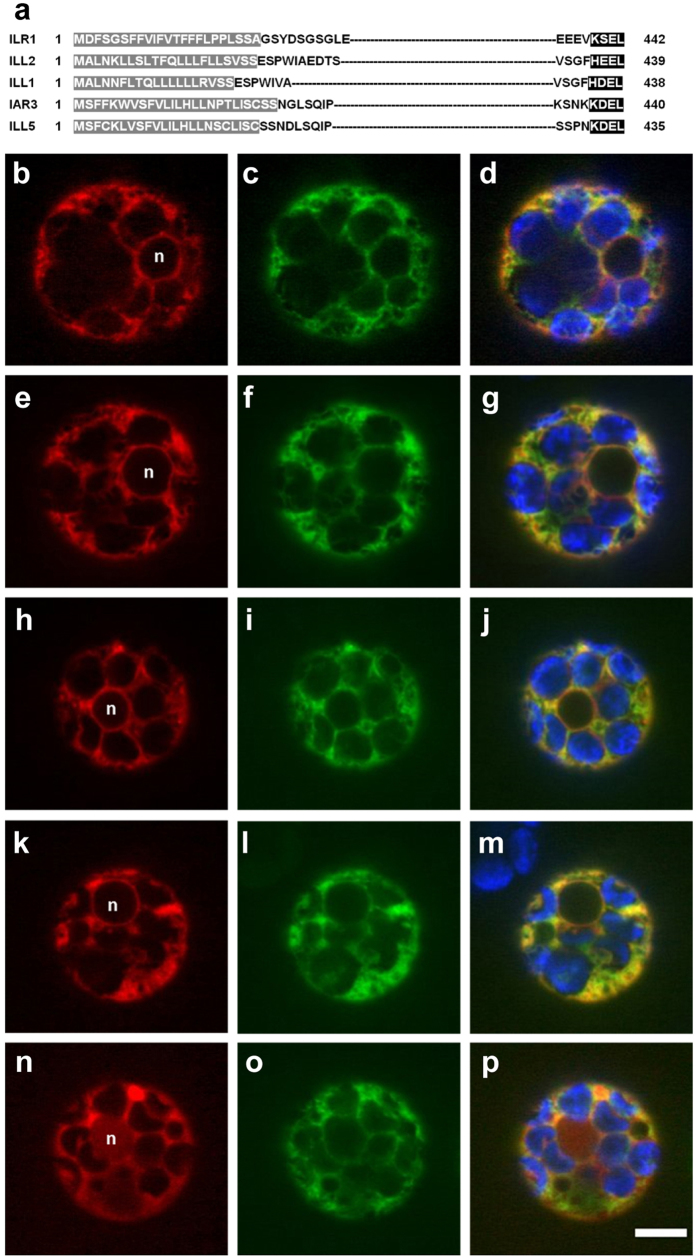
Subcellular localization of ILR1, ILL2 and IAR3. (**a**) N- and C-terminal protein sequences of Arabidopsis ILR1, ILL2, IAR3, ILL1 and ILL5. The predicted signal peptides, according to TargetP^40^, are highlighted in grey; the potential retention signals are highlighted in black. (**b–j**) Subcellular localization of ILR1, ILL2 and IAR3. Confocal images of Arabidopsis leaf protoplasts co-expressing the mCherry-HDEL ER marker (**b,e,h**) and either ILR1 (**c**) ILL2 (**f**) or IAR3 (**i**) fused to eGFP. (**k–p**) Co-localization analysis of ILL2 depleted of the potential retention signal HEEL (**l**) with the ER mCherry marker (**k**) or ILR1 depleted of the potential retention signal KSEL (**n**) with ILR1 fused to eGFP (**o**). Merged signal of red and green channels (**d,g,j,m,p**) indicates co-localization. In the merged images the chloroplasts are visualized in blue and in the red channel images the nucleus position is indicated (**n**). The scale bar corresponds to 5 μM.

We next analyzed in what cellular compartment the hydrolases function. Except for ILL6 and ILL3, the other five members of the ILR1-like family have both a predicted amino terminal signal sequence and a carboxy terminal peptide that might serve as an ER retrieval tag^24^. In particular, ILL1, IAR3 and ILL5 have a K/HDEL carboxy-terminal peptide (Fig. 4a), a well-known ER retention signal for soluble proteins^25^. ILR1 and ILL2 have the variant motives (KSEL and HEEL, respectively), for which there is no evidence of ER retention in plants. Based on these *in silico* analysis, it has long been assumed that some of these hydrolases are ER-localized. This, in combination with the relatively recent characterization of auxin carriers localized at the ER^26^,^27^,^28^, has led to the suggestion of a possible role of the ER in the conjugation-based metabolism of auxin^29^,^30^. However, the subcellular localization of the ILR1-like amidohydrolases has not been validated until now. We therefore decided to experimentally test the *in silico* predictions. We generated translational fusions of IAR3, ILL2 and ILR1 with the fluorescent protein GFP inserted at the N-terminal end of the proteins after the predicted signal peptide (Fig. 4a). The fusion proteins were transiently co-expressed with an ER-mCherry marker in Arabidopsis protoplasts and visualized using spinning disk confocal microscopy (Fig. 4b–j). The overlap of the fluorescent signals of ILR1-GFP, ILL2 and IAR3-GFP with the ER-mCherry marker confirmed the ER localization of the ILR1-like amidohydrolases (Fig. 4d,g,j). We further studied the *in silico* predicted ER retention signals and showed that the KSEL motif is essential for the retention of ILR1 in the ER. Upon removal of the tetrapeptide, the mutated ILR1-mCherry protein showed a cytoplasm-like localization and could be detected in the nucleus (Fig. 4n). On the other hand, removal of HEEL from ILL2 (Fig. 4l) did not affect the localization pattern suggesting that other motives/interactions are sufficient to retain ILL2 in the ER.

All together our findings indicate that in Arabidopsis hydrolysis of amide conjugates with auxin-like activity indeed occurs in the ER. These results provide first experimental evidence to a current model of subcellular compartmentation of auxin metabolism^1^, according to which conjugate hydrolysis takes place preferably in the ER, whilst the synthesis of IAA-aa conjugates is most likely happening in the cytosol. Besides Arabidopsis, auxin conjugate hydrolases from other species have ER retention sequences. Hydrolases with a typical retention signal have been characterized in *Brassica rapa*^31^. Additionally, phylogenetic analysis of ILR1-like family orthologues across the plant kingdom^32^ identified two monocot clades that evolved from the dicot orthologues: one possessing putative ER localization signals and one lacking them. The inverse reaction, the synthesis of IAA-aa conjugates, is catalyzed by members of the GH3 family^33^. So far, GH3 subcellular location has been studied only in moss *Physcomitrella patens*, where GH3-1 was experimentally shown to be cytoplasmic and GH3-2 was predicted to be in the cytosol^34^. Therefore, further experimental data are needed to understand the interplay between the ER and the cytosol located proteins involved in synthesis and hydrolysis of amide-linked IAA-l-amino acids conjugates.

Direct impact of IAA-aa hydrolysis on activation of auxin signaling suggests that the ER is not only a site of auxin storage. Gain of function mutants of ER localized auxin carriers PIN5, PIN8 and PIL5 resulted in altered auxin response^26^,^27^,^28^ supporting the idea that the IAA content in the ER indeed plays an important role in auxin signaling. Recently, enzymes involved in auxin biosynthesis and catalysing adjacent steps in YUCCA-dependent biosynthesis were also shown to be localized to the ER and this led to the suggestion that the relative concentration of auxin in different subcellular compartments might represent an additional cellular strategy to regulate auxin action^35^. Interestingly, IAR3 is also involved in the hydrolysis of jasmonyl-L-isoleucine (JA-Ile), which is the critical molecule to activate the jasmonate pathway^11^. Thus, the ER might also represent a site of metabolic/signaling crosstalk between auxin and jasmonate pathways.

## Methods

### Plant material

*Arabidopsis thaliana* wild type (Col-0) and amidohydrolase single and triple mutants *ilr1*, *iar3* and *ilr1ill2iar3*^20^ plant cultures were as described in^14^. *ilr1*, *iar3* and *ilr1ill2iar3* seeds were kindly provided by Dr. Bethany Zolman.

### Constructs

Total RNA isolated from cauline leaves or inflorescence of Arabidopsis thaliana Col-0 was used as template to amplify the cDNAs of *ILL2* (AT5G56660)*, ILR1* (AT3G02875) and *IAR3* (AT1G51760) using the primer pairs ILL2 for/ILL2 rev, ILR1 for/ILR1 rev and IAR3 for/IAR3 rev, respectively. The obtained fragments were cloned in the pENTR/D-TOPO plasmid (Invitrogen, Germany) to generate the plasmids pENTR-ILL2, pENTR-ILR1 and pENTR-IAR3. pENTR-ILR1 was used as template to amplify the clone ILR1-wo-KSEL using the primer pairs ILR1 for/ILR1woKSEL rev. The CDS ILR1woKSEL was further cloned in the pENTR/D-TOPO plasmid. Mistake-free clones were subsequently introduced by Gateway cloning into the pMIR vector containing the L2 min17-Luc auxin sensor and an additional 35S driven expression cassette^14^ to generate the plasmids: pMIR-L2 min17-Luc-ILL2, pMIR-L2 min17-Luc-ILR1and pMIR-L2 min17-Luc-IAR3. Control auxin sensor plasmid containing the GFP expression cassette was used according to^14^. Fluorescently tagged ILL2, ILR1 and IAR3 were generated by translational fusion with eGFP (GenBank AFA52654.1), with eGFP inserted after the amino acid position 33, 34 and 30 from the start codon (ATG), for ILL2, ILR1 and IAR3, respectively. GFP was inserted using the Gibson assembly method. To assemble the plasmid pENTR-ILL2eGFP, three PCR fragments were combined: one amplified with the primers ILL2-eGFP for/eGFP-ILL2 rev and eGFP’s cDNA as template; one amplified with the primers eGFP-ILL2 for/pENTR Kan and pENTR-ILL2 as template; and one amplified with the primers pENTR Kan for/ILL2-eGFP rev and pENTR-ILL2 as template. To assemble the plasmid pENTR-ILR1eGFP, three PCR fragments were combined: one amplified with the primers ILR1-eGFP for/eGFP-ILR1 rev and eGFP’s cDNA as template; one amplified with the primers eGFP-ILR1 for/pENTR Kan and pENTR-ILR1 as template; and one amplified with the primers pENTR Kan for/ILR1-eGFP rev and pENTR-ILR1 as template. To assemble the plasmid pENTR-ILR1eGFPwoKSEL, the same primers employed to generate the clone pENTR-ILR1eGFP were used but pENTR-ILR1woKSEL was used as PCR template instead of pENTR -ILR1. To assemble the plasmid pENTR-IAR3eGFP, three PCR fragments were combined: one amplified with the primers IAR3-eGFP for/eGFP-IAR3 rev and eGFP’s cDNA as template; one amplified with the primers eGFP-IAR3 for/pENTR Kan and pENTR-IAR3 as template; and one amplified with the primers pENTR Kan for/IAR3eGFP rev and pENTR/D-TOPO-IAR3 as template. To assemble the plasmid pENTR-ILL2eGFPwoHEEL, three PCR fragments were combined: one amplified with the primers ILL2-eGFP for/ILL2woHEEL rev; one amplified with the primers ILL2woHEEL for/pENTR Kan rev; and one amplified with the primers pENTR Kan for/ILL2-eGFP rev. For all the three fragments pENTR-ILL2eGFP was used as PCR template. pENTR-ILL2eGFP, pENTR-ILR1eGFP, pENTR-IAR3eGFP, pENTR-ILL2eGFPwoHEEL and pENTR-ILR1eGFPwoKSEL were subsequently used for Gateway cloning with the plant expression vector p2GW7.0^36^. The sequences of all the primers used are given in Table 1.

### Gene expression analysis

For expression analysis of auxin induced genes in Arabidopsis seedlings, 10-day old Col-0 and *ilr1ill2iar3* seedlings were transferred to liquid SCA medium^37^ supplied with 10 μM each of IAA-Ala, IAA-Leu, IAA-Phe or 100 nM IAA. After 1 hour, 3–4 seedlings/condition were frozen in liquid nitrogen and further used for total RNA isolation. For expression analysis of ILR1-like genes in Arabidopsis Col-0 protoplasts and for expression analysis of *ILR1*, *ILL2* and *IAR3* in transiently transformed *ilr1ill2iar3* protoplasts, 5∙10^5^cells/sample were harvested 20 h after isolation/transformation, and used for total RNA isolation. Total RNA isolation was performed with RNeasy Plant mini kit (Qiagen, Germany) according to the manufacture instructions, with an additional DNA elimination step. 250 ng of purified RNA was used for cDNA synthesis with Maxima Reverse Transcriptase (Fermentas, Germany) using a combination of oligo(dT) and random primers and following the manufacture instructions. For RT-PCR analysis, 1 μl for each reaction was used for subsequent PCR amplification using the DreamTaq polymerase (Fermentas, Germany) according to the manufacture instructions. 25 amplification cycles were used. Equal amount of each reactions were loaded on 2% agarose gel. For qPCR analysis, DyNAmo Flash SYBR Green qPCR Kit (Thermo Scientific, Germany) was used. Real-time was performed using LightCycler 480 (Roche, Germany). For expression analysis of auxin induced genes, relative quantification analysis was applied. For expression analysis of ILR1, ILL2 and IAR3 in transient transformed protoplast, absolute quantification using a standard curve was applied. Primer pairs used for RT-PCR and qPCR are given in Table 1.

### Protein expression analysis

For protein accumulation analysis of ILR1, ILL2 and IAR3, triple mutant protoplasts were transformed using the plasmids p2GW7-ILR1eGFP, ILL2eGFP and IAR3eGFP, respectively. 20 h after transformation, 1*10^6^ cells were collected and used for microsomal enriched protein isolation according to^38^. Membrane pellets were suspended in 50 μl sample buffer^38^. Half sample was used for Western analysis, another half for Coomassie staining. Gels were blotted, stained and Western analysis was performed using standard procedures. For Western analysis GFP antibody (Roche) was used. Chemi-luminescent signal was recorded using Fusion Capt Advance SL4 software (Fusion SL, Peqlab).

### Protoplast isolation, transformation and luminescence analysis

For transient expression assays and for luminescence measurement assays using the auxin sensor, isolation of Arabidopsis protoplasts, PEG-mediated DNA uptake, auxin treatments and luminescence measurements were performed as described in^14^. For each luminescent measurement, ~1.8*10^4^ protoplasts/sample were used. For each trial 6–8 samples were analysed. Data are presented as average of two independent trials and a total of 12–16 samples. The ratiometric values were used for statistical analysis performed using one-way ANOVA method in Minitab software (Minitab, Ltd, UK). Grouping was performed using Tukey’s multiple comparisons with 95% simultaneous confidence intervals. Statistical significances are indicated with lower case letters, means that do not share a letter are significantly different. Auxin treatment was performed 19-20 hours after DNA transfection, for 1 hour. IAA (Dushefa Biochemie, The Netherlands) was prepared as 10 mg/ml stock; IAA-L-Phe (Aldrich^CPR^, Germany MAR000005), IAA-L-Leu (Santa Cruz Biotechnologie, Germany CAS 57105-39-2), IAA-L-Ala (Santa Cruz Biotechnologie, Germany sc-257809) were prepared as 10 mM stock. All stocks were prepared in 90% Ethanol and stored at −20 °C.

For subcellular localization analysis the following plasmids were used: p2GW7.0-ILL2eGFP, p2GW7.0-ILR1eGFP, p2GW7.0-ILR1woKSELmCherry, p2GW7.0-ILL2woHEELeGFP, p2GW7.0-IAR3eGFP and ER-mCherry^39^.

### Microscopy

Spinning disk Andromeda microscope (Till Photonics GmbH, Germany) was used in the analysis of the subcellular localization of the fluorescently tagged amidohydrolases and the ER compartment. Image acquisition was performed 20 h after protoplast transformation using LA software (Till Photonics GmbH, Germany). The GFP-tagged proteins were excited with 488 nm diode laser and detected with BP 525/50 emission filter. The mCherry-tagged proteins were excited with 561 nm diode laser and detected with BP 593/40 emission filter. Chlorophyll fluorescence of the chloroplasts was detected using 488 nm diode laser for excitation and quad-band BP FF01-446/523/600/677-25 filter for emission. Images were acquired as Z-stacks with 0.35 nm step size using 63xNA 1.3 Immersion-water objective (Carl Zeiss, Jena). Representative confocal sections through the nucleus area were used for image processing of the detected channels using Fiji version of ImageJ software (http://fiji.sc/Fiji).

## Additional Information

**How to cite this article**: Sanchez, A. P. *et al.* Hydrolases of the ILR1-like family of Arabidopsis thaliana modulate auxin response by regulating auxin homeostasis in the endoplasmic reticulum. *Sci. Rep.* **6**, 24212; doi: 10.1038/srep24212 (2016).

## Supplementary Material

Supplementary Figure S1

## Figures and Tables

**Table 1:** Oligonucleotide sequences.

Primer name	Sequence (5’ to 3’)	Use
ILL2 for	CACCATGGCTCTAAACAAGCTCCTCAGT	cDNA amplification
ILL2 rev	TTAGAGTTCTTCATGAAAGCCTGA	
IAR3 for	CACCAGAGATAAGTCATGAGTTTCTTC	
IAR3 rev	AGAAGCCAATGTTTGTTGGCA	
ILR1 for	CACCATGGATTTCTCAGGGAGCTTCT	
ILR1 rev	AGCTGATTTTCTCCCAACACC	
RT-IAR3 for	AAGTGAGCTCGAGAGAGGGT	RT-PCR/qPCR
RT-IAR3 rev	CATATTCACGCTCGCTTGCC	
RT-ILL1 for	TTGGTGGCATCGGTTGGTTA	
RT-ILL1 rev	CGTGAAACTGACCCTTCGGA	
RT-ILL2 for	AAGGGAATCCGGGCAGAAAG	
RT-ILL2 rev	CATGCTTGCGGACATGATGG	
RT-ILL3 for	TGCTCCTTGGTGCTGCTAAA	
RT-ILL3 rev	CCAAAGCAGGACCCGAGATT	
RT-ILL5 for	AGGATCCACGAGAACCCAGA	
RT-ILL5 rev	CTCAAAGCAACAAAGGGGGC	
RT-ILL6 for	CTTTGGATGACGTGGAGGCT	
RT-ILL6 rev	GCGCCACATCGAGACTATGA	
RT-ILR1 for	CACGGTTCACGGTCAAGGT	
RT-ILR1 rev	ACCGATGCTTGTGCCTCTG	
RT-IAA1 for	ATGGAAGTCACCAATGGGCTTAACCT	
RT-IAA1 rev	TCATAAGGCAGTAGGAGGAGCTTCGGATC	
RT-IAA5 for	TCCGCTCTGCAAATTCTGTTCGG	
RT-IAA5 rev	CCCAAGGAACATCTCCAGCAAG	
RT-SAUR15 for	ATGGCTTTTTTGAGGAGTTTCTTGGG	
RT-SAUR15 rev	TCATTGTATCTGAGATGTGACTGTG	
RT-SAUR66 for	CACAAAGAAACTCATGAAGATGG	
RT-SAUR66 rev	GAATCGAATCGAATGGCAAC	
RT-GH3.5 for	AGCCCTAACGAGACCATCCT	
RT-GH3.5 rev	AAGCCATGGATGGTATGAGC	
RT-ACT for	TGCTGGACGTGACCTTACTG	
RT-ACT rev	TCTCGATGGAAGAGCTGGT	
ILR1-eGFP for	GGTTCGGGTCTCGAGTCAGTGAGCAAGGGCGAGGAGCTGT	Cloning
eGFP-ILR1 rev	CCCGCGAGCGAGGCCGCCCTTGTACAGCTCGTCCATGCCG	
eGFP-ILR1 for	GACGAGCTGTACAAGGGCGGCCTCGCTCGCGGGATGCTTCATTC	
pENTR Kan rev	CCATACAAGCGATAGATTGTCG	
pENTR Kan for	ATAATGTCGGGCAATCAGGTG	
ILR1-eGFP rev	CTCCTCGCCCTTGCTCACTGACTCGAGACCCGAACCAGAA	
IAR3-eGFP for	TCTAATGGGTTATCTCAAGTGAGCAAGGGCGAGGAGCTGT	
eGFP-IAR3 rev	CTTTGAAGGTATGCCGCCCTTGTACAGCTCGTCCATGCCG	
eGFP-IAR3 for	GAGCTGTACAAGGGCGGCATACCTTCAAAGTTTCTTACTT	
IAR3-eGFP rev	CTCCTCGCCCTTGCTCACTTGAGATAACCCATTAGAGGAA	
ILL2-eGFP for	GCCGAAGATACGTCTCAAGTGAGCAAGGGCGAGGAGCTGT	
eGFP-ILL2 rev	CTTCGTCTGGATGCCGCCCTTGTACAGCTCGTCCATGCCG	
eGFP-ILL2 for	GAGCTGTACAAGGGCGGCATCCAGACGAAGCTCCTCGAAT	
ILL2-eGFP rev	CTCCTCGCCCTTGCTCACTTGAGACGTATCTTCGGCGATC	
ILR1woKSEL rev	CTAAACCTCTTCTTCATGGCTATGAC	
ILL2woHEEL for	GTCTCAGGCTTTTAACGACCCAGCTTTCTTGTACAAAGTT	
ILL2woHEEL rev	CAAGAAAGCTGGGTCGTTAAAAGCCTGAGACAGAACCTTTAG	
