# Supplementary material for: Hydrolases of the ILR1-like family of Arabidopsis thaliana modulate auxin response by regulating auxin homeostasis in the endoplasmic reticulum
Source: Sci Rep. 2016 Apr 11;6:24212. doi: 10.1038/srep24212 (PMC4827090; doi:10.1038/srep24212)
Supplement: Supplementary Figure S1 [file srep24212-s1.pdf]

Supplementary information

Hydrolases of the ILR1-like family of Arabidopsis thaliana modulate auxin response by regulating auxin homeostasis in the endoplasmic reticulum

Ana Paula Sanchez[a+], Aparajita Singh[a+], Karoline Steinberger[a], Kishore Panigrahi[b], Klaus Palme[a,c,d,e], Alexander Dovzhenko[a], Cristina Dal Bosco[a*]

[+] These authors contributed equally

[a]Institute of Biology II/Molecular Plant Physiology, Faculty of Biology, Albert-Ludwigs-University of Freiburg, Schänzlestrasse 1, D-79104 Freiburg, Germany;

[b]National Institute of Science Education and Research, Institute of Physics Campus, Bhubaneswar , Odisha 751005, India.

[c] BIOSS Centre for Biological Signalling Studies, University of Freiburg, 79104 Freiburg, Germany;

[d] Freiburg Institute for Advanced Sciences (FRIAS), University of Freiburg, 79104 Freiburg, Germany;

[e] Centre for Biological Systems Analysis (ZBSA), University of Freiburg, 79104 Freiburg, Germany.

*Cristina Dal Bosco (E-mail: dal.bosco.cristi@gmail.com)

Supplementary Fig. S1

Expression analysis of ILR1, ILL2 and IAR3 in transiently transformed Arabidopsis protoplasts

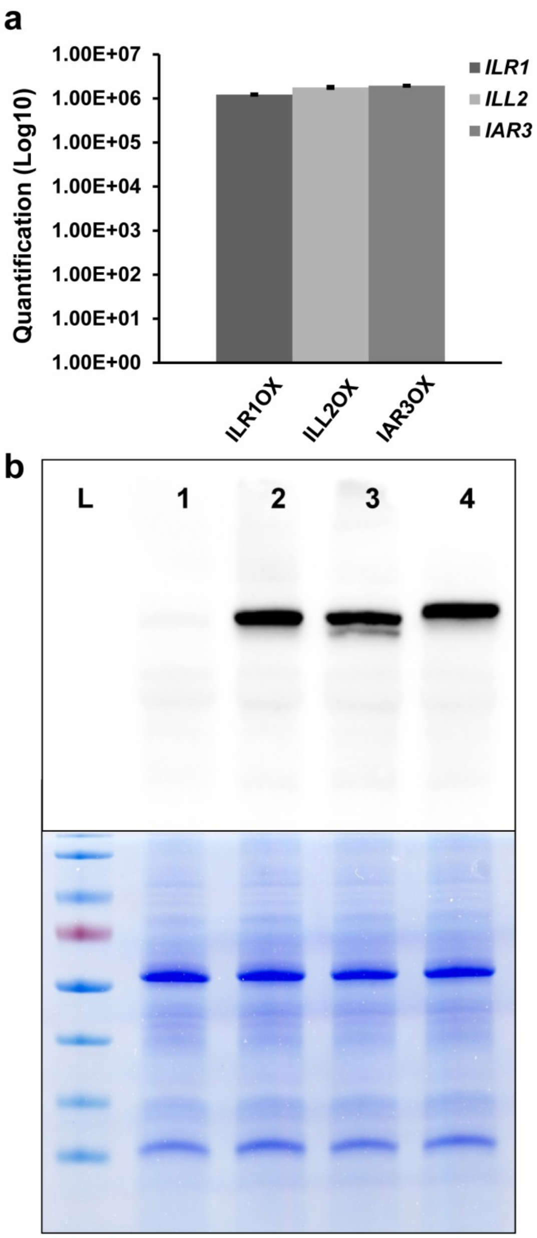

Supplementary Fig. S1

Expression analysis of ILR1, ILL2 and IAR3 in transiently transformed Arabidopsis protoplasts. (a) Absolute quantification of *ILR1*, *ILL2* and *IAR3* transcripts in *ilr1ill2iar3* cells. Transformations were performed with the sensor construct harboring cassettes with *ILR1* (ILR1OX), *ILL2* (ILL2OX) and *IAR3* (IAR3OX), respectively. The quantification was performed using standard curves. Transcript quantities are expressed as copies /10 ng of total RNA. (b) Western blot analysis and Coomassie staining of proteins prepared from transiently transformed protoplasts expressing ILR1eGFP (2), ILL2eGFP (3) and IAR3eGFP (4). The blot was immunodecorated with anti-GFP antibody. Untransformed protoplasts (1) served as negative control.
